# Pretreatment with atorvastatin ameliorates cobra venom factor-induced acute lung inflammation in mice

**DOI:** 10.1186/s12890-020-01307-3

**Published:** 2020-10-12

**Authors:** Jing Guo, Min Li, Yi Yang, Lin Zhang, Li-wei Zhang, Qian-yun Sun

**Affiliations:** 1 State Key Laboratory of Functions and Applications of Medicinal Plants, Guizhou Medical University, Guiyang, 550014 China; 2 Center for Pharmacology and Bioactivity Research, The Key Laboratory of Chemistry for Natural Products of Guizhou Province and Chinese Academy of Sciences, Guiyang, 550014 China; 3 Modern Research Center for Traditional Chinese Medicine, Shanxi University, Taiyuan, 030006 China; 4 General Ward, Guizhou Provincial People’s Hospital, Guiyang, 550002 China; 5 Key Laboratory of Chemical Biology and Molecular Engineering of Education Ministry, Institute of Molecular Science, Shanxi University, Taiyuan, 030006 China

**Keywords:** Atorvastatin, Acute lung inflammation, Complement, Cobra venom factor, Aspirin

## Abstract

**Background:**

The complement system plays a critical role as the pathogenic factor in the models of acute lung injury due to various causes. Cobra venom factor (CVF) is a commonly used complement research tool. The CVF can cause acute inflammation in the lung by producing complement activation components. Atorvastatin (ATR) is a 3-hydroxy-3-methylglutaryl coenzyme A inhibitor approved for control of plasma cholesterol levels. This inhibitor can reduce the acute pulmonary inflammatory response. However, the ability of ATR in treating acute lung inflammation caused by complement activation is still unknown. Therefore, we investigated the effect of ATR on lung inflammation in mice induced by activation of the complement alternative pathway in this study.

**Methods:**

ATR (10 mg/kg/day via oral gavage) was administered for 7 days before tail vein injection of CVF (25 μg/kg). On the seventh day, all mice were sacrificed 1 h after injection. The lung lobe, bronchoalveolar lavage fluid (BALF), and blood samples were collected. The myeloperoxidase (MPO) activity of the lung homogenate, the leukocyte cell count, and the protein content of BALF were measured. The levels of interleukin-6 (IL-6), tumor necrosis factor-α (TNF-α), P-selectin, and Intercellular cell adhesion molecule-1 (ICAM-1) in BALF and serum were determined by enzyme-linked immunosorbent assay. The pathological change of the lung tissue was observed by hematoxylin and eosin staining. The deposition of C5b-9 in the lung tissue was detected by immunohistochemistry. The phosphorylation of NF-κB p65 in the lung tissues was examined by immunohistochemistry and western blotting.

**Results:**

The lung inflammation levels were determined by measuring the leukocyte cell numbers and protein content of BALF, the lung MPO activity, and expression and staining of the inflammatory mediators (IL-6 and TNF-α), and adhesion molecules (P-selectin and ICAM-1) for lung lesion. A significant reduction in the lung inflammation levels was observed after 7 days in ATR pre-treated mice with a CVF-induced lung disease. Deposition of C5b-9 was significantly alleviated by ATR pretreatment. Early intervention with ATR significantly reduced the development of acute lung inflammation on the basis of phosphorylation of NF-κB p65 in the lung.

**Conclusion:**

These findings suggest the identification of ATR treatment for the lung inflammation induced by activating the complement system on the basis of its anti-inflammatory response. Together with the model replicating the complement activating characteristics of acute lung injury, the results may be translatable to the overactivated complement relevant diseases.

## Background

Acute lung injury (ALI) and its severe manifestation of acute respiratory distress syndrome (ARDS) have been a major problem in humans; this condition is characterized by acute inflammation and damage of microvascular endothelial cells in the early stages [1]. Clinical [2, 3] and experimental studies [4, 5] have suggested the important role of complement activation products in the pathophysiology of ALI. In the early development of injury, the complement system is first activated; the abnormal or excessive activation of the complement system exhibits a startup and amplification effect in the initial stages of the inflammatory response [6, 7]. The complement system plays a critical role as the pathogenic factor in the models of acute lung injury due to various causes [8–11]. On this basis, we choose cobra venom factor (CVF), a commonly used complement research tool, to induce rapid pulmonary inflammation for exploring the pathologic changes and effective medications of lung damage. This work is conducted to truly reflect the status of pulmonary inflammation in vivo.

CVF is the protein from cobra venom that can activate complement [12]. The complement activating pattern of CVF is highly in accord with the way of complement activation in the pathological conditions of the body owing to the homology to complement component C3 in structure and function [12, 13]. The CVF, as a complement experimental tool, has been extensively used to clarify its involvement in the pathogenesis of numerous diseases, including acute lung inflammation [14–17]. A previous study [17] has shown that an intravenous (i.v.) injection of CVF activated the complement alternative pathway that leads to the production of early response cytokines, such as tumor necrosis factor-α (TNF-α) and interleukin-6 (IL-6). CVF triggers a powerful pro-inflammatory cascade that eventually leads to acute injury of the lung.

Despite the great advances in the research and therapy, ALI/ARDS mortality is still high, and therapies are scarce [18]. Statins, which are the inhibitors of 3-hydroxy-3-methylglutaryl coenzyme A (HMG-CoA), are used to control the blood cholesterol levels. Recent articles [19–21] have demonstrated multipotent activities of statins, such as anti-inflammatory and anti-oxidant activities, in addition to their lipid-lowering properties; clinical evidence shows the decreasing effects of lung inflammation of statins [22, 23]. Experimental studies have shown that atorvastatin (ATR) could reverse lung injury and decrease the alveolar infiltration and pro-inflammatory mediator production in the initial stage of a variety of lung damage animal models [1, 22, 24]. However, the study of ATR on acute lung inflammation caused by complement activation remains unclear. In this model, the objective was to elucidate the effectiveness and mechanism of ATR treatment on an acute lung inflammation model induced by activation of the complement alternative pathway.

## Methods

### Reagents

CVF was isolated from the venom of *Naja atra* by our laboratory group in a previous study [25]. ATR was purchased from Jialin Pharmaceutical Incorporated Company (Beijing, China). ASA was purchased from Yunnan Baiyao Incorporated Company (Kunming, China). Carboxymethylcellulose sodium (CMC-Na) was obtained from Shanghai Aladdin Biochemical Technology Incorporated Company (Shanghai, China). The myeloperoxidase (MPO) kit was bought from Nanjing Jiancheng Bioengineering Institute (Nanjing, China). The BCA protein assay kit was obtained from Beyotime Biotechnology Co., Ltd. (Shanghai, China). Enzyme-linked immunosorbent assay (ELISA) kits for determination of mouse IL-6, TNF-α, P-selectin, and ICAM-1 were obtained from Boster Biological Technology Co., Ltd. (Wuhan, China). A rabbit anti-C5b-9 antibody was purchased from Abcam (Cambridge, MA, USA). Antibodies for Ser 311-phosphorylated NF-κB p65 and β-actin were obtained from Santa Cruz Biotechnology (Paso Robles, CA, USA).

### Animals

Thirty two Kunming mice (body weight 16 ± 2 g; 3–5 weeks old; half male and half female) were provided by Hunan SJA Laboratory (Changsha, Hunan, China). Before the experiment, the animals were fed standard rodent chow and water and monitored in a controlled temperature and under a 12 h light/dark cycle for 5 days. The experiments were performed in accordance with the protocols approved by the Institutional Animal Care and Use Committee of Guizhou Medical University (No. 1800239).

### Experimental design and mouse model of ALI

The CVF at a dose of 25 μg/kg body weight in sterile phosphate buffered saline (PBS, pH 7.4) was administered via tail vein injection to specifically activate the complement alternative pathway as in previous studies [17, 26]. This step was carried out to establish the mouse model of ALI. The control group received PBS. Before inducing the model, the animals were orally pretreated with ASA (aspirin 200 mg/kg/day [27], as a positive control) or ATR (10 mg/kg/day [21, 28]) prepared in 0.5% CMC-Na for 7 days. The vehicle-treated control group received equal volume of 0.5% CMC-Na. On the seventh day, mice were sacrificed by an intraperitoneal (i.p.) injection of pentobarbital sodium (50 mg/kg) 1 h after CVF or PBS challenge. Blood samples were collected in tubes via retroorbital bleed for biochemical analyses. The blood samples were left at room temperature for 1 h to induce clotting and centrifuged at 3000 r/min for 10 min at 4 °C to obtain serum. The serum was stored at − 80 °C until used in the study.

**Fig. 1:**
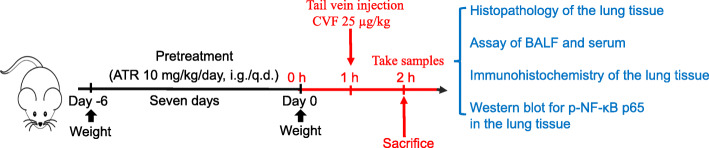
Experimental timeline of the study design. Mice were weighed and grouped at day − 6. Subsequently, the mice were orally pretreated with ASA (aspirin, 200 mg/kg/day), ATR (atorvastatin, 10 mg/kg/day), or an equal volume of 0.5% CMC-Na once daily (q.d.) for 7 days until day 0. On day 0, the mice were weighed and given with ASA, ATR, or 0.5% CMC-Na via the intragastric route 1 h prior to the induction of acute lung inflammation via CVF injection (tail vein, i.v.). The control group received PBS injection. The mice (*n* = 8/each group) were sacrificed 1 h after CVF injection, and the blood, BALF, and lung tissues were collected for index measurement. CMC-Na, carboxymethylcellulose sodium; CVF, cobra venom factor; PBS, phosphate buffered saline; BALF, bronchoalveolar lavage fluid

A median sternotomy was allowed for exposure of both of the lungs. After the hilum of the right lung was ligated, the trachea was exposed and inserted with a lavage needle. Thereafter, the left lung was lavaged for four times with 0.3 ml ice-cold saline to obtain the bronchoalveolar lavage fluid (BALF). The BALF from each sample was centrifuged at 3000 r/min and 4 °C for 10 min to pellet the cells after the protein content of BALF was checked with the BCA assay kit. The supernatant was stored at − 80 °C for cytokine measurement, and the resuspended cell pellet was counted with a hemocytometer. The superior lobe of the right lung was rapidly removed and stored at − 80 °C until use. The middle lobe of the right lung was excised for the analysis of lung wet/dry weight ratio. The lower lobe of the right lung was fixed in 4% paraformaldehyde for histopathologic and immunohistochemical examination (Fig. 1).

### Lung wet/dry weight ratio

The middle lobe of the right lung was cut off and weighed as wet weight, and the lung was placed in a 70 °C incubator for at least 48 h to acquire the dry weight [18]. The ratio of wet to dry lung was calculated.

$$ \mathrm{Lung}\ \mathrm{W}/\mathrm{D}\ \mathrm{ratio}\ \left(\%\right)=\left(\mathrm{wet}\ \mathrm{weight}-\mathrm{dry}\ \mathrm{weight}\right)/\mathrm{wet}\ \mathrm{weight}\times 100\% $$

### MPO activity

The lung tissues were thawed and homogenized in normal saline. The tissue homogenate was tested for the MPO level (an indicator of neutrophil accumulation [29]) with a test kit. All procedures were followed in accordance with the manufacturer’s instructions.

### Assay of BALF and serum

The levels of IL-6, TNF-α, P-selectin, and ICAM-1 in BALF and serum were determined with ELISA kits. Meanwhile, the content of protein in BALF was estimated with the BCA protein assay kit. Total leukocyte counts were conducted under a microscope (Nikon, Tokyo, Japan) with Neubauer chambers.

### Histopathological examination

The lower lobe of the right lung was dehydrated, embedded in paraffin, and cut into 5 μm sections. The sections were stained with hematoxylin–eosin (H&E) and evaluated under an optical microscope. The pathological severity score of the lung was evaluated by an investigator, who was blinded to the grouping, as previously described [30]. The scoring contents included the following four criteria: alveolar congestion, hemorrhage, infiltration of leukocytes in the lung tissue, and thickness of the alveolar wall/hyaline membrane formation. The score range was 0–4 points (0, minimal damage or very mild; 1, 2, 3 and 4 represented mild, moderate, severe and maximal damage, respectively.). Points were added up and the mean of total scores for the four indicators was calculated as the lung injury score.

### Immunohistochemistry

The streptavidin–biotin complex method was used to test C5b-9 and the phosphorylation of NF-κB p65 in the 5 μm lung sections. The lung tissue sections were stained with rabbit antibody against C5b-9 (1:100) and phospho-NF-κB p65 (1:500) overnight at 4 °C and incubated with horseradish peroxidase-conjugated goat anti-rabbit IgG antibody (Biosynthesis Biotechnology, Beijing, China) for 1 h at room temperature. Finally, the lung sections were stained in brown using diaminobenzidine (Beyotime Biotechnology, Shanghai, China) and then counterstained with hematoxylin. The phosphorylation level of NF-κB p65 was detected according to the average optical density by using Image-Pro Plus software. Scoring for C5b-9 staining was performed on the lung tissues using a 0–3 points scoring system: 0 represented no staining, and 1, 2, and 3 denoted low, moderate, and high staining, respectively.

### Western blot analysis

The tissues were homogenized, and the protein concentrations were determined with the BCA protein assay kit. The proteins (30 μg) were separated on a denaturing 10% polyacrylamide gel and transferred to a polyvinylidene difluoride membrane. The following primary antibody was used: phospho-NF-κB p65 (1:1000). The membranes were then incubated with the secondary antibody at a dilution of 1:1000 (goat anti-rabbit IgG) for 1 h at room temperature and imaged with the Vilber Fusion FX6 Spectra imaging system. The bands were quantified using VisionCapt v16.15 software (Vilber Lourmat, France). β-Actin (1:1000) was used as an internal control.

### Statistical analysis

Statistical analyses of data were performed using SPSS 18.0. Data were expressed as mean ± standard error of the mean (SEM). For lung injury scores, total cells in BALF, ICAM-1 in serum and C5b-9 deposition statistical analysis were assessed with the Mann-Whitney U test. Other data analysis was conducted by using one-way analysis of variance. *P* < 0.05 between two groups was considered statistically significant.

## Results

### Histopathological findings

**Fig. 2:**
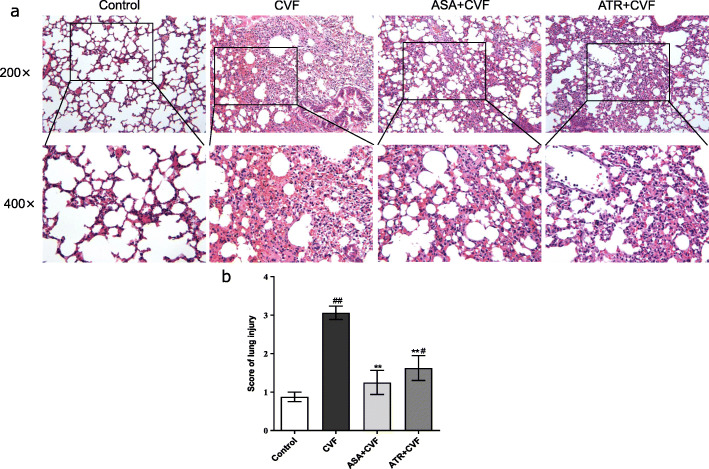
ATR ameliorated the CVF-induced acute lung inflammation in mice. The tissue morphology was analyzed 1 h after CVF intravenous administration. The four independent parameters were observed to evaluate the pathological severity response of the lungs: alveolar congestion, hemorrhage, infiltration of leukocytes in the lung tissue, and thickness of the alveolar wall/hyaline membrane formation. **a** Representative H&E sections of the lung. The original magnifications are × 200 and × 400. **b** Lung injury score. Results are presented as mean ± SEM (*n* = 8 in each group). ^#^*P* < 0.05 vs. the control group; _##_*P* < 0.01 vs. the control group; ^**^P < 0.01 vs. the CVF group. ATR, atorvastatin; CVF, cobra venom factor; ASA, aspirin; H&E, hematoxylin and eosin

We investigated the effect of ATR on the lung histopathologic change in mice with CVF challenge (Fig. 2). The histological analysis revealed that the lung injury of CVF-challenged mice was characterized by alveolar septal edema, mild expansion, congestion, and inflammatory cell infiltration. ASA and ATR pretreatment alleviated these changes. However, pretreatment with ATR had no significant difference compared with that with ASA. The results demonstrated that ATR attenuated CVF-induced lung inflammation.

### Effect of ATR on neutrophil infiltration of lungs

**Fig. 3:**
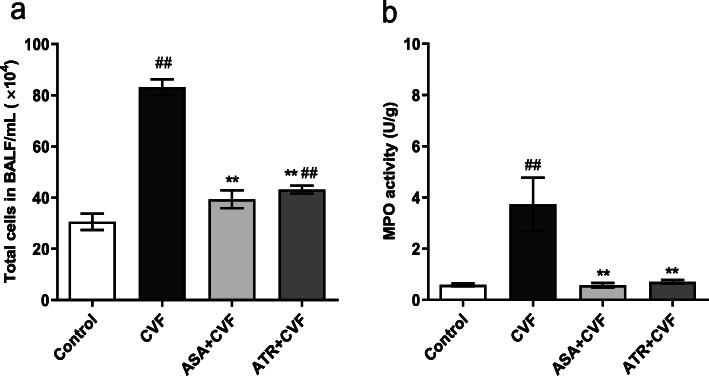
ATR reduced the cell counts in the BALF and inhibited neutrophil infiltration in the lung tissues following CVF challenge. The cell counts were assessed with a hemocytometer. The superior lobe of the right lung was excised to detect the lung MPO activity 1 h after the CVF challenge. **a** Total cells in the BALF. **b** Lung MPO activity. Results are presented as mean ± SEM (*n* = 8 in each group). ^#^*P* < 0.05 vs. the control group; ^##^*P* < 0.01 vs. the control group; ^**^P < 0.01 vs. the CVF group. ATR, atorvastatin; BALF, bronchoalveolar lavage fluid; ASA, aspirin; CVF, cobra venom factor; MPO, myeloperoxidase; SEM, standard error of the mean

The accumulation of activated neutrophils in the lung is the first step in the pulmonary inflammation that leads to acute lung injury and histological damage. The total leukocyte cell counts in BALF and the MPO activity in the lung tissues were detected to investigate neutrophil activation. Figure 3a and b demonstrate that a marked increase of 63.15 and 84.22% in the total cell counts in BALF and MPO in the lung homogenate, respectively, was observed in the CVF-challenged mice compared with that in the control mice (*P* < 0.01). The ATR pretreatment significantly decreased the total cell counts and MPO level following CVF challenge to 48.12 and 81.02% (*P* < 0.01), respectively. The increases in mice pretreatment with ASA after CVF administration were 52.63 and 84.49% compared with those in the CVF-challenged mice.

### Effect of ATR on the total protein level of BALF and wet to dry weight ratio of lungs

**Fig. 4:**
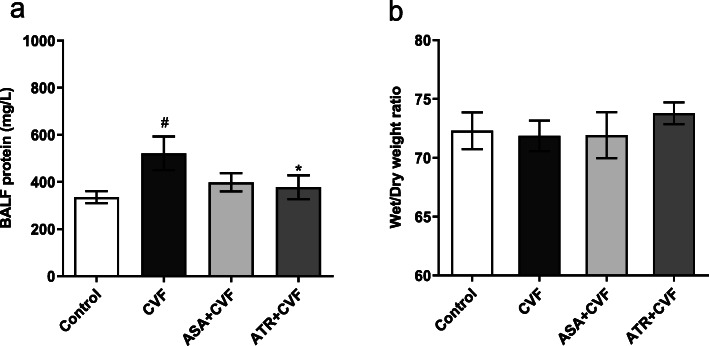
ATR suppressed the total protein level of BALF and wet to dry weight ratio of lungs. **a** Total protein contents in the BALF. **b** Lung wet to dry weight ratio (W/D) in the middle lobe of the right lung. Results were presented as mean ± SEM (*n* = 8 in each group). ^##^*P* < 0.01 vs. the control group; ^*^P < 0.05 vs. the CVF group. ATR, atorvastatin; BALF, bronchoalveolar lavage fluid; CVF, cobra venom factor; SEM, standard error of the mean

Figure 4a illustrates that the CVF group markedly increased the protein level compared with the control group (*P* < 0.05). The pretreatment with ATR significantly reduced the protein concentration in BALF compared with the CVF group (*P* < 0.05). Figure 4b shows the effect of different pretreatments on the wet to dry weight ratio of lungs. However, all groups showed no statistically significant differences.

### Effect of ATR on the inflammatory mediators and adhesion molecules in BALF

**Fig. 5:**
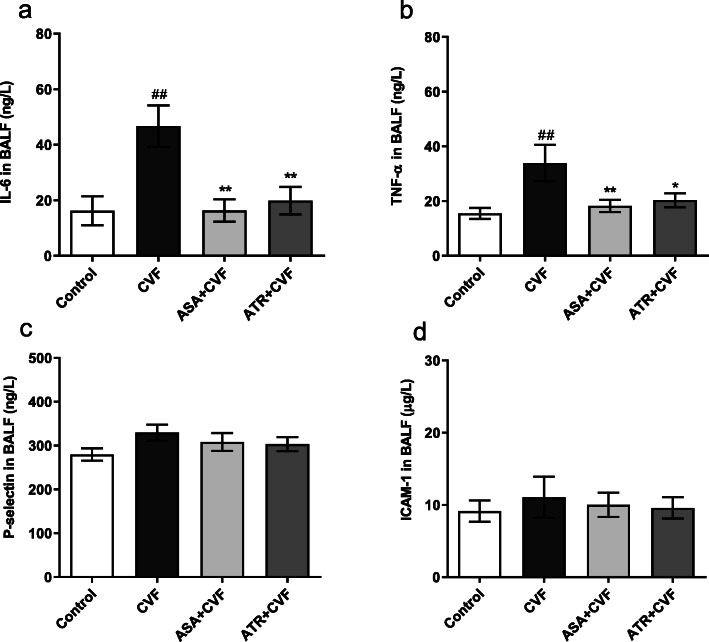
ATR suppressed the inflammatory mediators and adhesion molecules in BALF. The inflammatory mediators (IL-6 and TNF-α) and adhesion molecules (ICAM-1 and P-selectin) were assessed with ELISA kits. Levels of (**a**) IL-6, (**b**) TNF-α, (**c**) P-selectin, and (**d**) ICAM-1 in BALF. Results were presented as mean ± SEM (*n* = 8 in each group). ^##^*P* < 0.01 vs. the control group; ^*^P < 0.05 vs. the CVF group; ^**^P < 0.01 vs. the CVF group. ATR, atorvastatin; BALF, bronchoalveolar lavage fluid; IL-6, interleukin-6; TNF-α, tumor necrosis factor-α; ICAM-1, intercellular cell adhesion molecule-1; ELISA, enzyme-linked immunosorbent assay; CVF, cobra venom factor; SEM, standard error of the mean

Figure 5a and b show that the levels of IL-6 and TNF-α in the CVF group were significantly increased by 65.23 and 54.29%, respectively, compared with those in the mice of the control group (*P* < 0.01). The pretreatment with ATR significantly reduced the levels of IL-6 and TNF-α in BALF by 57.47 and 40.11%, respectively, compared with those in the CVF group (*P* < 0.05 and 0.01). The levels of IL-6 and TNF-α in the pretreatment with ASA compared with that with ATR were reduced by 17.77 and 10.34%, respectively. No statistically significant differences in the levels of P-selectin and ICAM-1 were noted in BALF from the control, CVF, ATR + CVF, and ASA + CVF groups (Fig. 5c and d).

### Effect of ATR on the inflammatory mediators and adhesion molecules in serum

**Fig. 6:**
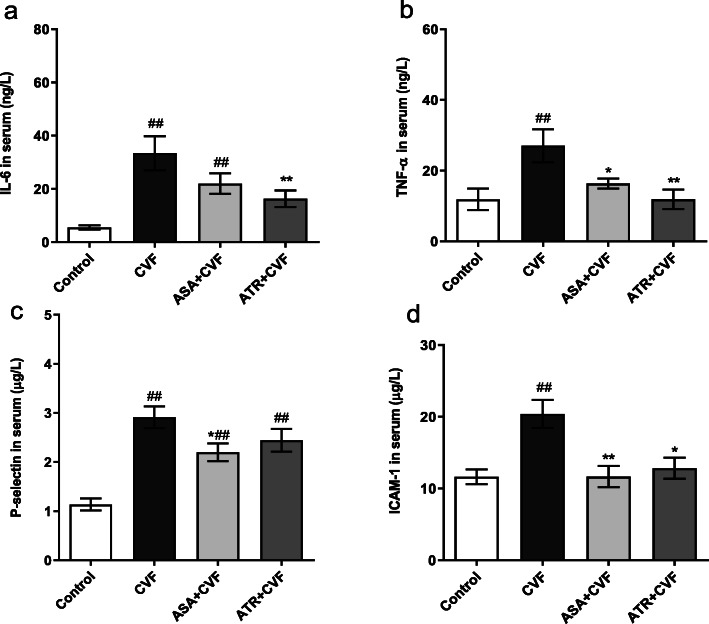
ATR suppressed the inflammatory mediators and adhesion molecules in serum. The inflammatory mediators (IL-6 and TNF-α) and adhesion molecules (ICAM-1 and P-selectin) were assessed with ELISA kits. Levels of (**a**) IL-6, (**b**) TNF-α, (**c**) P-selectin, and (**d**) ICAM-1 in serum. Results were presented as mean ± SEM (*n* = 8 in each group). ^#^*P* < 0.05 vs. the control group; ^##^*P* < 0.01 vs. the control group; ^*^P < 0.05 vs. the CVF group; ^**^P < 0.01 vs. the CVF group. ATR, atorvastatin; BALF, bronchoalveolar lavage fluid; ASA, aspirin; IL-6, interleukin-6; TNF-α, tumor necrosis factor-α; ICAM-1, intercellular cell adhesion molecule-1; ELISA, enzyme-linked immunosorbent assay; CVF, cobra venom factor; SEM, standard error of the mean

Figure 6a–d exhibit the effect of ATR on the levels of IL-6, TNF-α, P-selectin, and ICAM-1 in serum. The levels of IL-6, TNF-α, P-selectin, and ICAM-1 in the CVF group were significantly increased by 83.46, 56.07, 60.82, and 42.99%, respectively, compared with the control group (*P* < 0.01). The pretreatment with the ATR markedly reduced the levels of IL-6, TNF-α, and ICAM-1 in serum compared with that in the CVF group with decreases of 51.18, 55.99, and 37.11%, respectively (*P* < 0.05 and 0.01). The levels of IL-6 and TNF-α in the pretreatment with ASA compared with ATR were reduced by 25.75 and 27.32%, respectively. However, no significant differences were seen in the levels of IL-6, TNF-α, P-selectin, and ICAM-1 in the serum from the ATR + CVF and ASA + CVF groups (*P* > 0.05).

### Effect of ATR on C5b-9 deposition of the lung tissue in mice with ALI

**Fig. 7:**
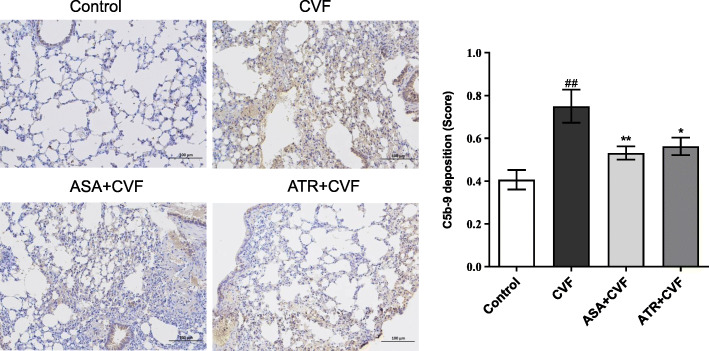
ATR suppressed the C5b-9 deposition of the lung tissue in mice with ALI. The deposition of C5b-9 was determined by immunohistochemistry. The original magnification was × 200. The C5b-9 deposition from the lung scores was shown as mean ± SEM (*n* = 7–8 in each group). ^##^*P* < 0.01 vs. the control group; ^*^P < 0.05 vs. the CVF group; ^**^P < 0.01 vs. the CVF group. ATR, atorvastatin; CVF, cobra venom factor; ASA, aspirin; ALI, acute lung injury; SEM, standard error of the mean

We examined the deposition of C5b-9 in the lung tissue to determine whether complement activation product C5b-9 was inhibited by ATR after CVF injection. The results showed that the deposition of C5b-9 significantly increased after CVF injection (*P* < 0.01) (Fig. 7). The C5b-9 deposition in the lung was significantly (*P* < 0.05) reduced by 25% in mice pretreated with ATR compared with CVF mice. Meanwhile, the score for C5b-9 deposition in mice pretreated with ASA was decreased by 29.17% compared with that in CVF mice. No significant differences on the C5b-9 deposition in the ATR + CVF group were observed compared with the ASA + CVF group (*P* > 0.05).

### Effect on NF-κB p65 phosphorylation in the lung tissue

**Fig. 8:**
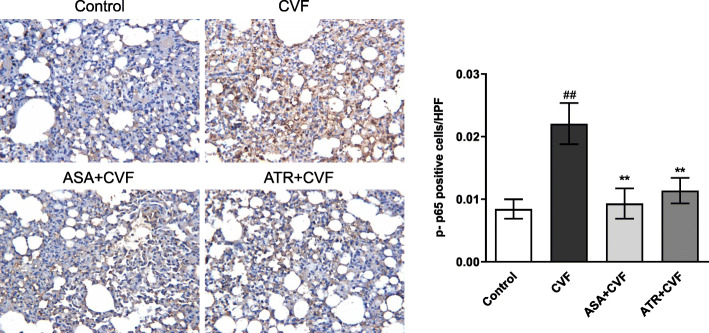
ATR inhibited the expression of phosphorylated NF-κB p65 in the lung tissue. The NF-κB p65 phosphorylation was determined by immunohistochemistr and the positive cells were marked by tan or brown. Results were semiquantitatively scored by averaging the number of stained cells per high-power field. The original magnification was × 400. Results were presented as mean ± SEM (*n* = 8 in each group). ^##^*P* < 0.01 vs. the control group; ^**^P < 0.01 vs. the CVF group. ATR, atorvastatin; CVF, cobra venom factor; ASA, aspirin; SEM, standard error of the mean

The NF-κB signal pathway plays an important role in the regulation of inflammation at the transcriptional level. The lung tissue sections were detected to locate phospho-NF-κB p65 in the lung tissue according to the positively stained cells by immunohistochemical method. This step was conducted to detect the effect of ATR on NF-κB. Figure 8 shows that stained cells in the lung tissue of the CVF group were positively and intensively increased by 62% compared with that in the control group. The ATR + CVF group showed fewer cells positively stained in the lung tissue with a decrease of 48.42% (*P* < 0.01) compared with the CVF group. No significant difference on the NF-κB p65 phosphorylation level in the ATR + CVF group was observed compared with that in the ASA + CVF group with a decrease of 57.92% (*P* > 0.05).

### Effect on phosphorylated NF-κB p65 protein expression in the lung tissue

**Fig. 9:**
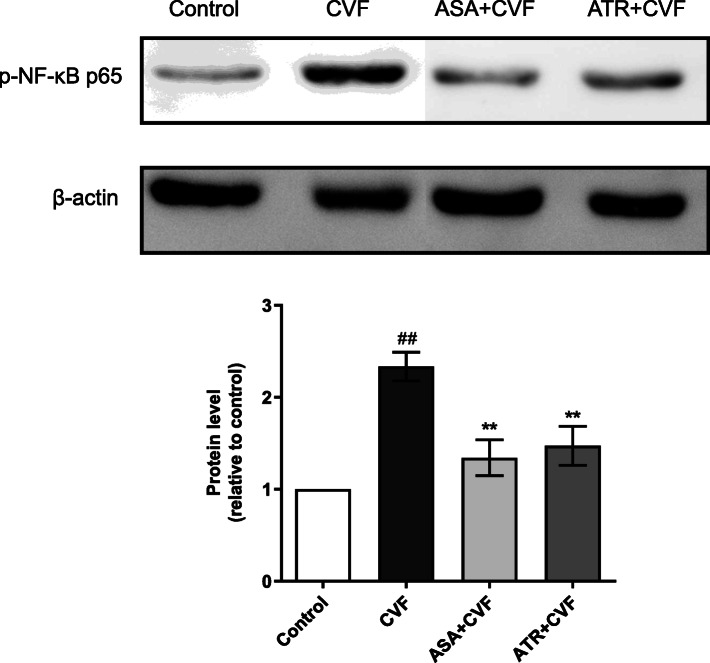
ATR inhibited NF-κB activation in the lung tissue of the CVF-induced lung inflammation mice. Phosphorylated NF-κB p65 protein expression was determined by western blot. The protein expression was normalized to β-actin. Thereafter, the protein/β-actin of the three groups (CVF, ASA + CVF, and ATR + CVF) was normalized to that of the control group. The protein blots were cropped on account of the presence of other groups. Results were presented as mean ± SEM (*n* = 8 in each group). ^##^*P* < 0.01 vs. the control group; ^**^P < 0.01 vs. the CVF group. ATR, atorvastatin; CVF, cobra venom factor; ASA, aspirin; SEM, standard error of the mean

The western blot images of phospho-NF-κB p65 and β-actin (housekeeping) proteins in different groups are presented in Fig. 9. Consistent with the result of immunohistochemical method, the CVF significantly increased the phospho-NF-κB p65 protein expression to 57.20% compared with the control mice (*P* < 0.01). The ATR + CVF group reduced the expression of phospho-NF-κB p65 (*P* < 0.01) with a decrease of 37.01% compared with the CVF group. No significant difference was observed in the phospho-NF-κB p65 protein expression of the ATR + CVF group compared with the ASA + CVF group with a decrease of 42.61% (*P* > 0.05). These data suggested that ATR may inhibit the inflammatory response by downregulating the NF-κB signal pathway activation.

## Discussion

Acute lung injury is a critical disease with a high incidence and mortality, and it is associated with pulmonary and systemic inflammation [23]. The complement system is one of the host defense mechanisms against infectious agents [31]. However, overactivation of the complement can lead to severe tissue damage, such as rheumatoid arthritis, sepsis, and ischemia reperfusion [6, 32]. The roles of complement in lung inflammatory injury are gradually identified [33]. Complement has been involved in the various models of acute lung injury [34–39]. Complement activation is an early step in ALI; the next complement activation products, such as C5a and/or the membrane attack complex (C5b-9), can activate the neutrophils to adhere to the pulmonary capillary endothelium and directly activate endothelial cells to increase P-selectin or work with TNF-α to make up for the upregulation of ICAM-1 and E-selectin [33, 40]. In the process, NF-κB, a transcription factor regulating various genes involved in the inflammatory and immune responses, is activated and triggers NF-κB p65 translocation to the nucleus. The augmented activation of NF-κB p65, a strong pro-inflammatory cascade, causes the transcription of inflammatory cytokines, such as TNF-α, IL-6, and IL-1β and further results in lung injury [41]. In the complement system, three pathways can initiate the process to lead to C3 activation: classical, alternative, and lectin [42]. However, the three activation pathways require engagement of the alternative pathway to cause tissue injury in vivo, including acute lung inflammation [43]. Hence, the excessive activation of complement alternative plays a crucial role in the occurrence and development of acute lung inflammation.

CVF is the protein from cobra venom that can specifically activate the complement alternative pathway. This protein, a structural and functional analog of complement component C3, is used to study the functions of complement in immune defense and in the pathogenesis of lung injury [12, 44]. CVF can bind to factor B, and the complex is then cleaved by factor D to form the C3/C5 convertase. Then, the convertase generates the potent pro-inflammatory anaphylatoxins C5a and C5b; the latter, together with C6–C9, forms the complement terminal component C5b-9 [45], which can result in cell lysis [46]. Herein, we choose CVF to study the development of acute lung inflammation by activating the complement alternative pathway of mice and successfully induced the lung inflammation model. In this study, the response to acute lung inflammation was assessed by the following factors: histopathological and immunohistochemical examinations, BALF and serum levels of inflammatory mediators (IL-6 and TNF-α) and adhesion molecules (P-selectin and ICAM-1), total leukocyte cells, content of protein in BALF, W/D weight ratio, MPO activity, and NF-κB p65 phosphorylation in the lung tissue. Tissue injury was histopathologically observed. The levels of the inflammatory mediators in BALF and serum were significantly increased after the induction of lung inflammation. The release of adhesion molecules in serum was markedly elevated. The phosphorylation of NF-κB p65 in the lung was notably upregulated in response to lung inflammation. A marked increase in the number of total cells in BALF and MPO activity in the lung homogenate and in the protein level of BALF were also observed. However, the W/D weight ratio of lungs showed no substantial change due to the unnoticeable pulmonary edema in the early stage of acute lung inflammatory lesions. In the meanwhile, complement terminal content C5b-9 of the lung tissue in the CVF-induced mice was significantly increased. These findings were consistent with our previous research [17]. In summary, this study found that the activation of the complement alternative pathway can induce acute lung inflammation in mice.

In the present study, we observed the effects of ATR on the lung inflammation model after activation of the complement alternative pathway in mice. Statins, which are inhibitors of 3-hydroxy-3-methylglutaryl coenzyme A reductase, were applied into clinic to lower cholesterol for minimizing cardiovascular events in the 1980s. Several publications describe the other applications of statins in anti-infective and immune modulations independent of lipid regulation [47]. The study of statins in relevant cellular and animals may also help understand the mechanism of endothelial dysfunction in acute pulmonary inflammation [23]. Jacobson et al. [48] reported that simvastatin could reduce the inflammatory parameters, such as MPO content in BALF, total neutrophil counts, and gene expression of NF-κB and IL-6 in LPS-mediated lung damage. Siempos et al. [1] demonstrated that pretreatment with ATR conferred protection against lung injury induced by high-stretch mechanical ventilation by attenuating alveolar capillary permeability. Li et al. [49] reported that simvastatin decreased the IL-6 level of the lung tissue in LPS-induced septic. Ren et al. [50] reported that ATR could alleviate the lung damage and reduce mortality rate in rats with sepsis. Although simvastatin is a common statin applied in acute lung injury, the anti-inflammatory effects demonstrate differences. Melo et al. [21] evaluated the effects of ATR, pravastatin, and simvastatin on endotoxin-induced ALI and showed that ATR and pravastatin but not simvastatin revealed anti-inflammatory activity. Pinho-Ribeiro et al. [51] evaluated the effects of ATR and simvastatin on mouse lung emphysema induced by cigarette smoke. The result indicated that ATR showed a better anti-inflammatory activity compared with simvastatin. The latter showed good anti-oxidant property. In combination with our search for all the studies that examined the role of statins in ARDS/ALI by China National Knowledge Internet (www.cnki.com) using the keywords (“atorvastatin” or “pravastatin”) and (“acute respiratory distress syndrome” or “acute lung injury” or “acute lung inflammation” or “ARDS” or “ALI”), ATR was often used to provide a protective effect for patients who have a risk of ARDS/ALI and the animal models of pulmonary inflammation from 2010 to 2019. However, the potential of ATR to attenuate lung inflammation due to the direct activation of complement is still unknown. Further studies of the therapeutic effects of ATR on acute lung inflammation are needed. In our study, CVF induced complement activation, inflammatory cytokine release, and adhesion molecules into the BALF and serum in addition to neutrophils and the other cells. By contrast, the ATR treatment antagonized the CVF-induced release of these mediators. The ATR pretreatment inhibited the phosphorylation of NF-κB p65 in the lung tissue and the deposition of C5b-9. Thus, these findings exhibited that ATR minimized the pathological lung injury induced by CVF.

We used ASA as a control drug to assess the efficacy of ATR. ASA, one of nonsteroidal anti-inflammatory drugs, has a definite anti-inflammatory effect and is used as an anti-inflammatory drug for acute and chronic inflammation. The preclinical [52–55] and clinical trials [56–61] have indicated that ASA plays a beneficial role in acute lung injury prevention and treatment. Our study indicated a similar result that pretreatment with ASA resulted in decreased pulmonary inflammatory lesions. No significant difference was observed between ASA and ATR pretreatment regarding these inflammatory outcomes.

The results showed that ATR plays a protective role against acute lung inflammation induced by activation of the complement alternative pathway. Our study extends the understanding of ATR in the development of ALI after tail vein injection of CVF. This finding indicates that ATR inhibits inflammatory cell infiltration and tissue impairment with the downregulation of adhesion molecule expression, pro-inflammatory cytokines, complement terminal product, and phosphorylation of NF-κB p65.

## Conclusions

This study further supports the therapeutic potential of ATR for the intervention of pulmonary inflammation. From the perspective of the complement pathogenesis, the report suggests the relationship among ATR, complement, and complement-induced diseases, such as acute lung inflammation, to a certain extent. Thus, ATR may be a novel therapeutic for complement-related diseases.

## Supplementary information

**Additional file 1.** Supplementary Information: Original western blot images.

## Data Availability

The datasets generated and/or analyzed during the current study are available from the corresponding author on a reasonable request.

## Abbreviations

CVF: cobra venom factor
ATR: atorvastatin
BALF: bronchoalveolar lavage fluid
ALI: acute lung injury
MPO: myeloperoxidase
IL-6: interleukin-6
TNF-α: tumor necrosis factor-α
ICAM-1: intercellular cell adhesion molecule-1
ARDS: acute respiratory distress syndrome
ASA: aspirin
H&E: hematoxylin and eosin
IgG: immunoglobulin G
