# Supplementary material for: Pretreatment with atorvastatin ameliorates cobra venom factor-induced acute lung inflammation in mice
Source: BMC Pulm Med. 2020 Oct 12;20:263. doi: 10.1186/s12890-020-01307-3 (PMC7552367; doi:10.1186/s12890-020-01307-3)
Supplement: Supplementary file 1 — Additional file 1. Supplementary Information: Original western blot images. [file 12890_2020_1307_MOESM1_ESM.docx]

**Supplementary Information: Original western blot images**

# We screened for the inhibitory effect of different compounds on acute lung inflammation induced by cobra venom factor in our study. The different compounds, including aspirin (ASA) and atorvastatin (ATR), inhibited NF-κB activation in lung tissue of cobra venom factor (CVF)-induced lung inflammation mice by determining phosphorylated NF-κB p65 protein expression using western blot. In this manuscript, we only calculated the results on ASA and ATR, and data of other compounds (PDTC, resveratrol, chlorogenic acid, and icariin) were not included. Due to the absence of samples, we only detected the protein content of 5 samples in Fig 1. In order to improve the transparency of the article, we provided all the full length original gel images (n = 8). The original images are shown below.

**Fig 1:**


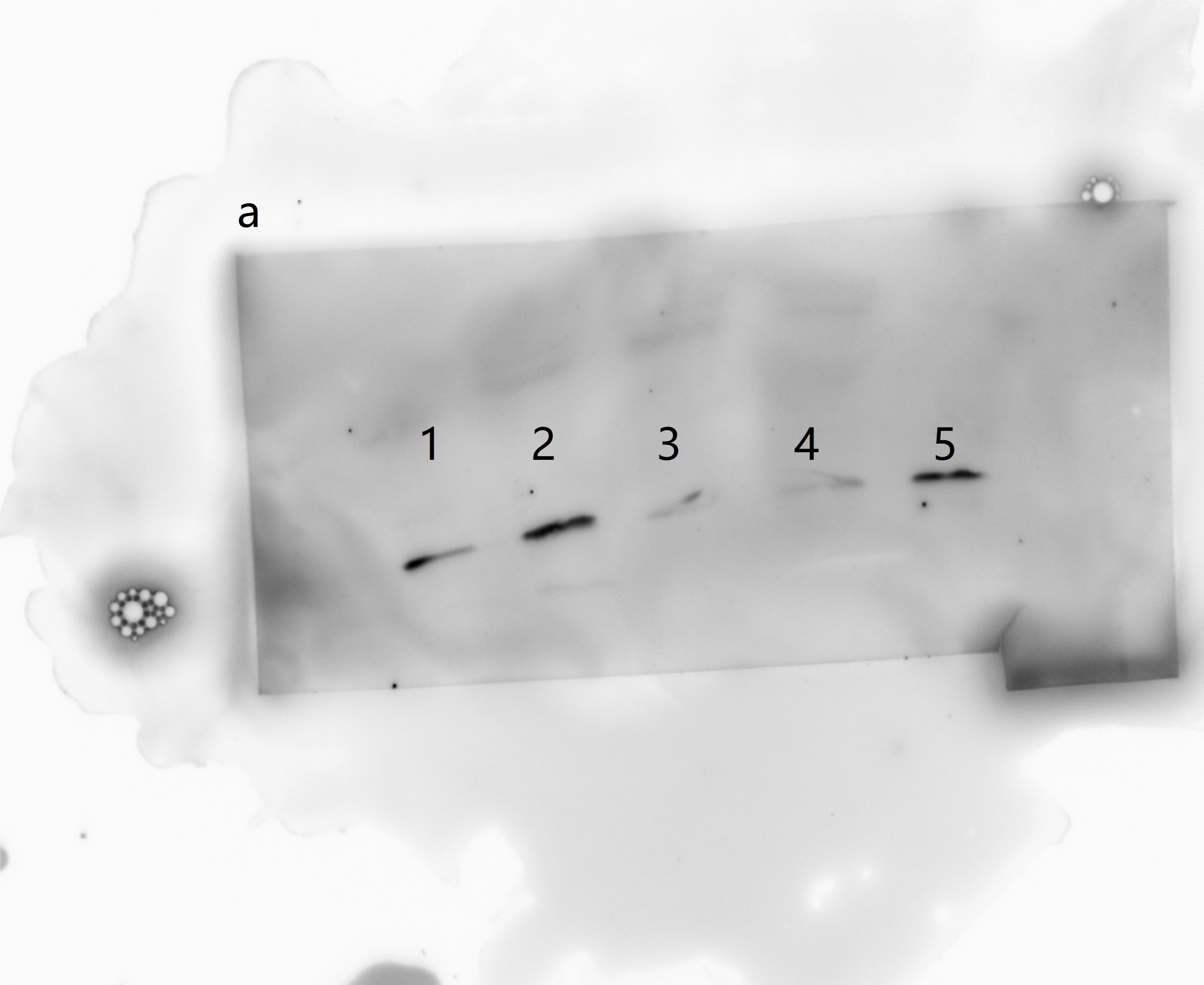


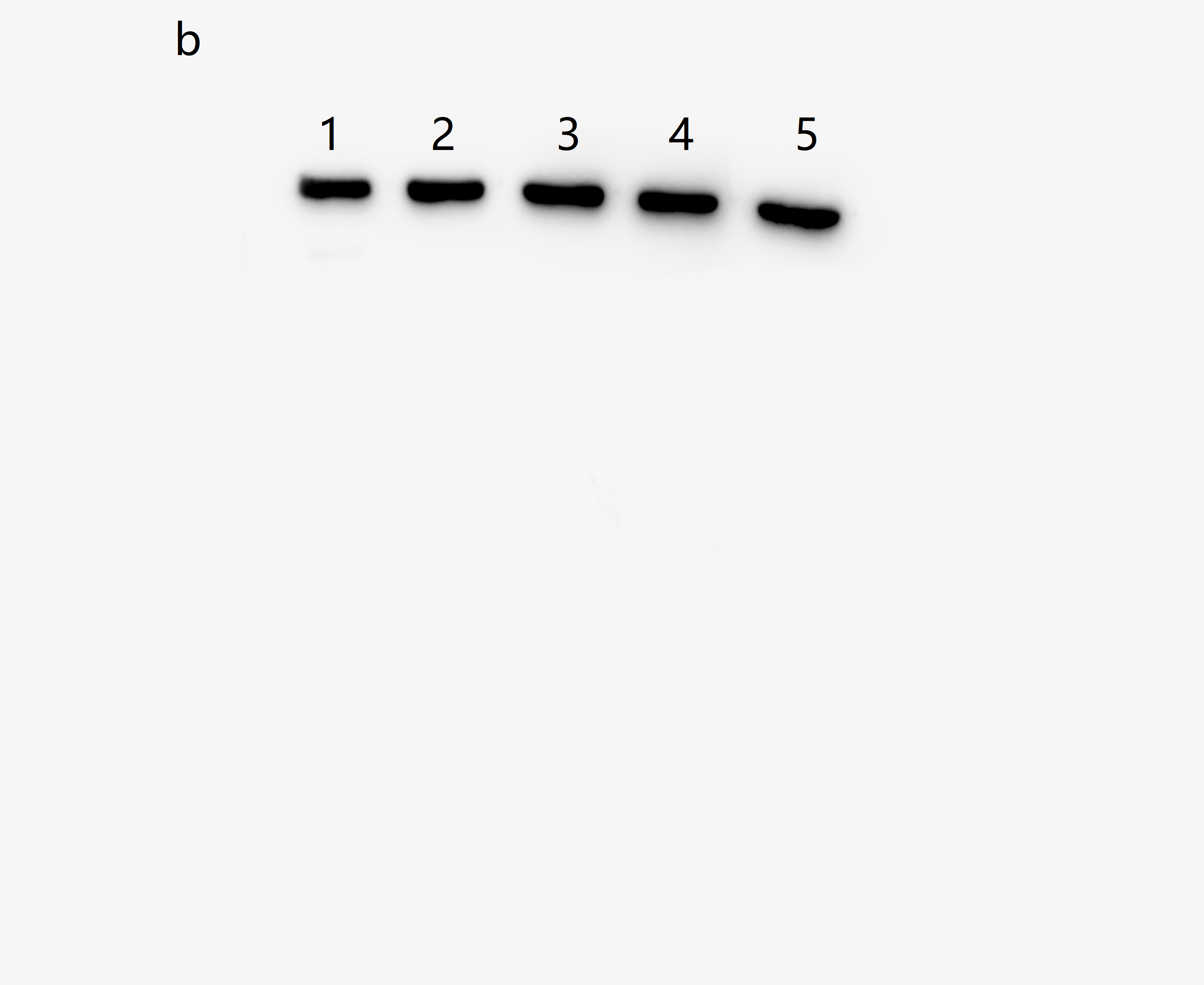


# **Fig 1.** The different compounds, including PDTC, aspirin (ASA) and atorvastatin (ATR), inhibited NF-κB activation in lung tissue of cobra venom factor (CVF)-induced lung inflammation mice by determining phosphorylated NF-κB p65 protein expression using western blot. Lanes 1-5: control, CVF, PDTC+CVF, ASA+CVF, ATR+CVF. (**a**) p-p65. (**b**) β-actin. In this manuscript, we only conducted the results on ASA and ATR. Data of PDTC was not included in this paper.

**Fig 2:**


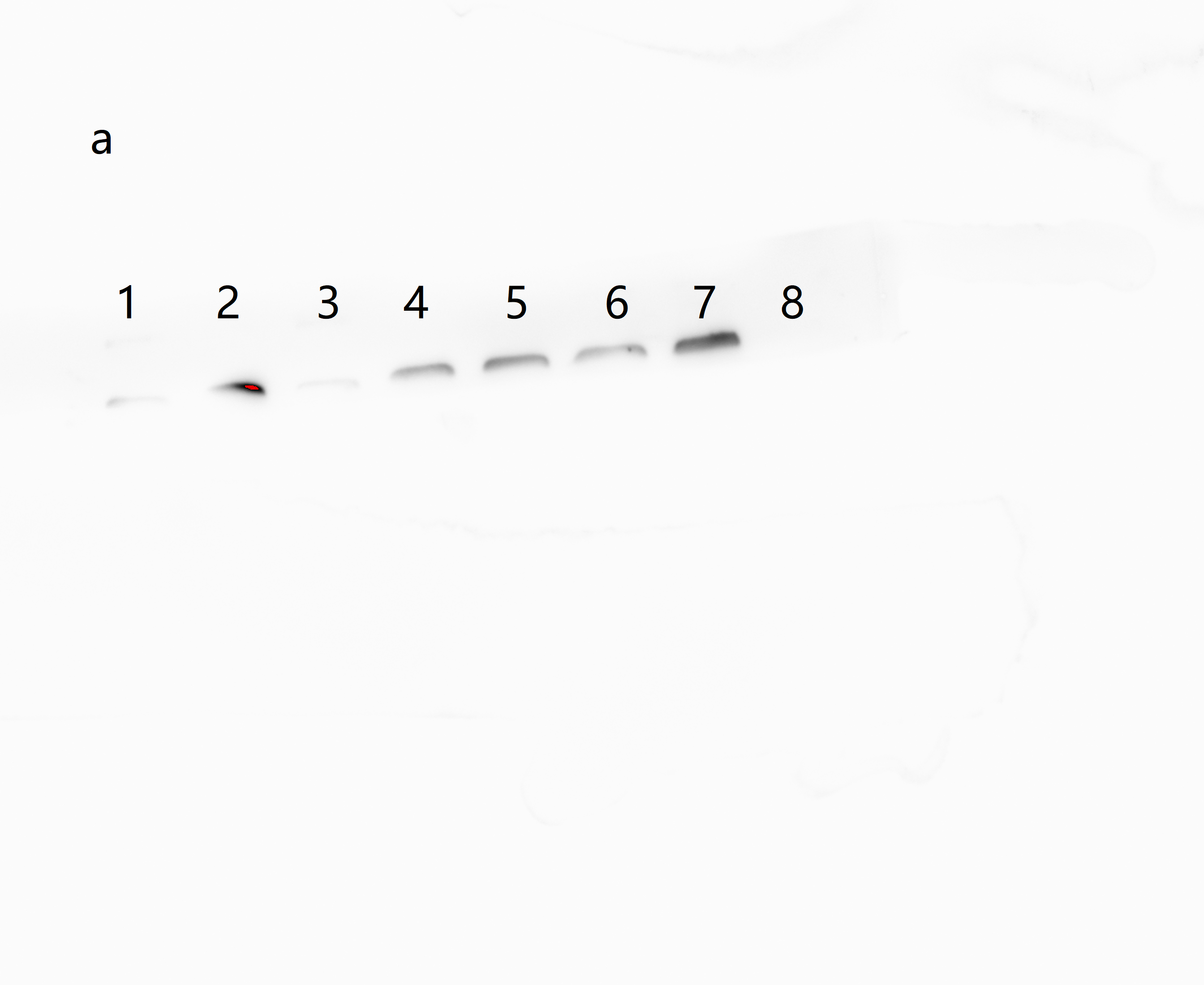

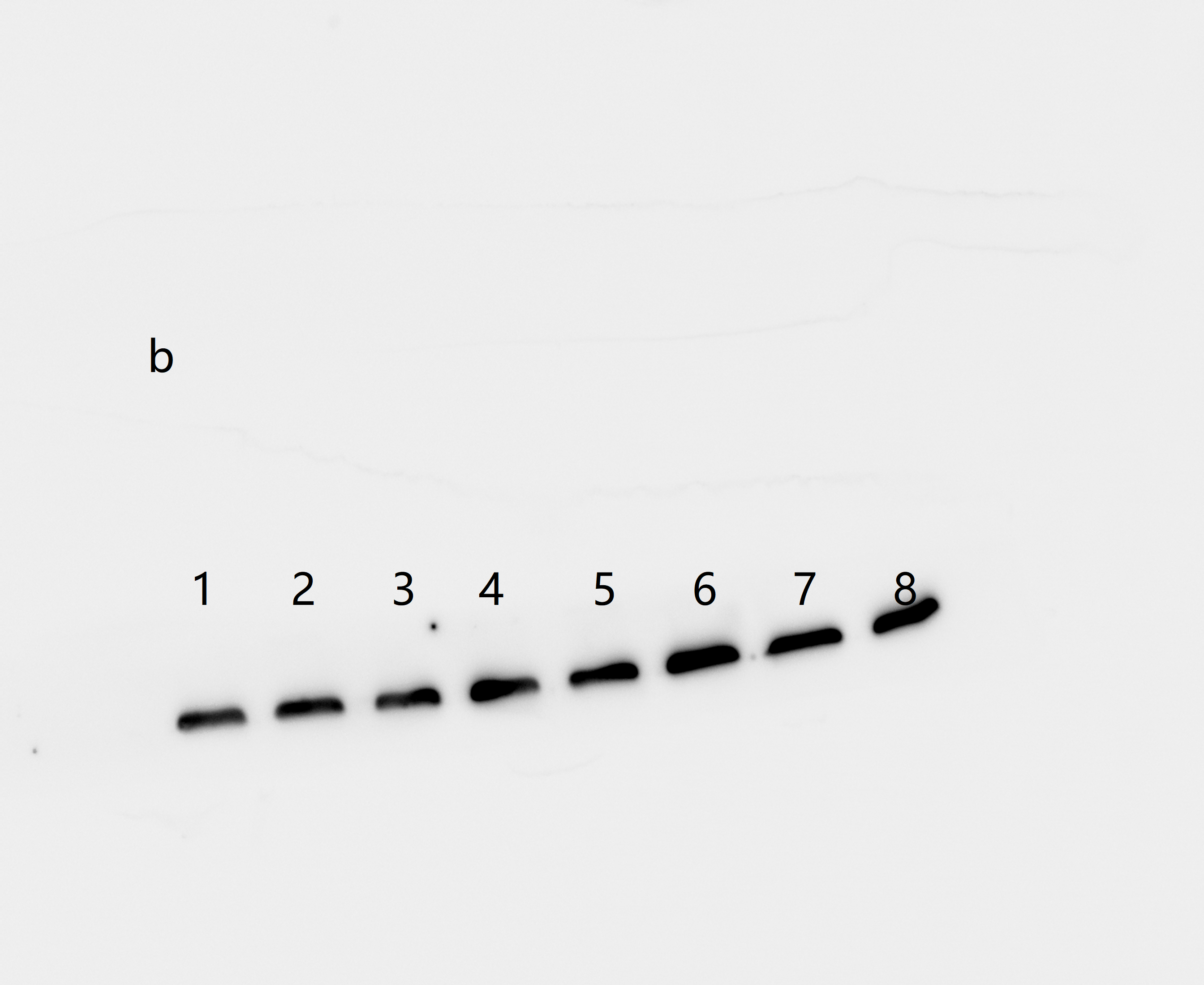


# **Fig 2.** The different compounds, including aspirin (ASA) and atorvastatin (ATR), inhibited NF-κB activation in lung tissue of CVF-induced lung inflammation mice by determining phosphorylated NF-κB p65 protein expression using western blot. Lanes 1-8: control, CVF, PDTC+CVF, ASA+CVF, ATR+CVF, resveratrol+CVF, chlorogenic acid+CVF, icariin+CVF. (**a**) p-p65. (**b**) β-actin. In this manuscript, we only conducted the results on ASA and ATR, and data of other compounds (PDTC, resveratrol, chlorogenic acid, and icariin) were not conducted.

**Fig 3:**


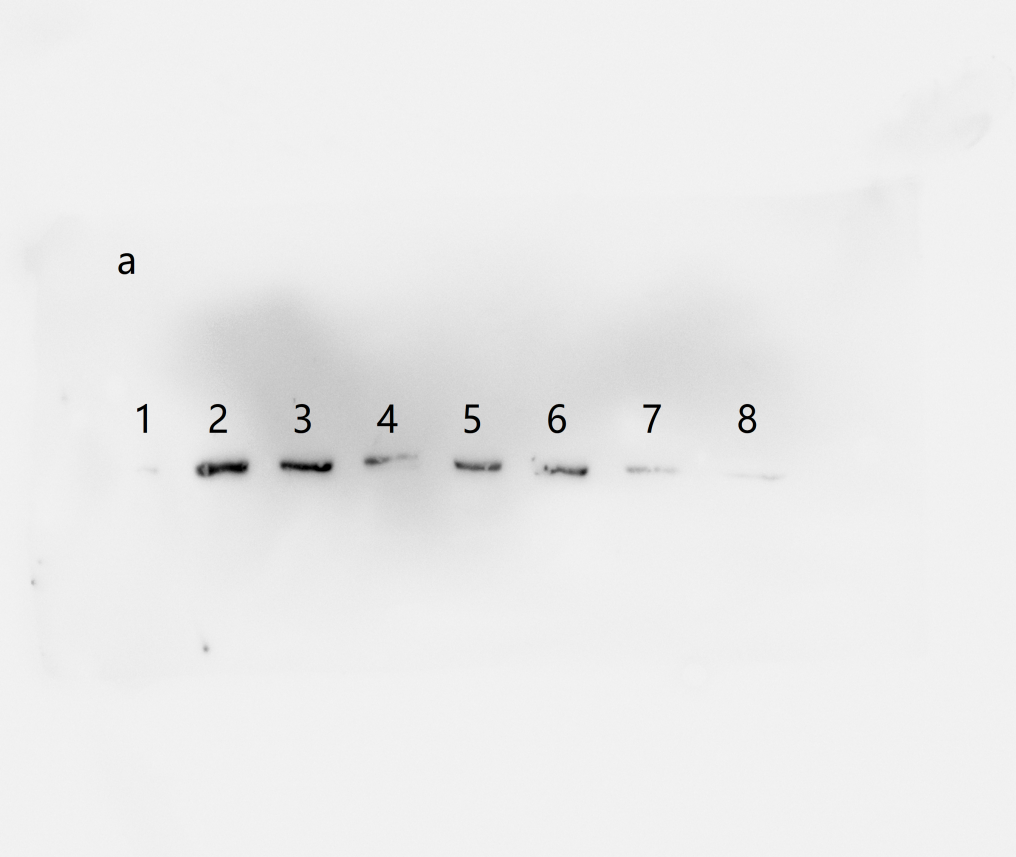


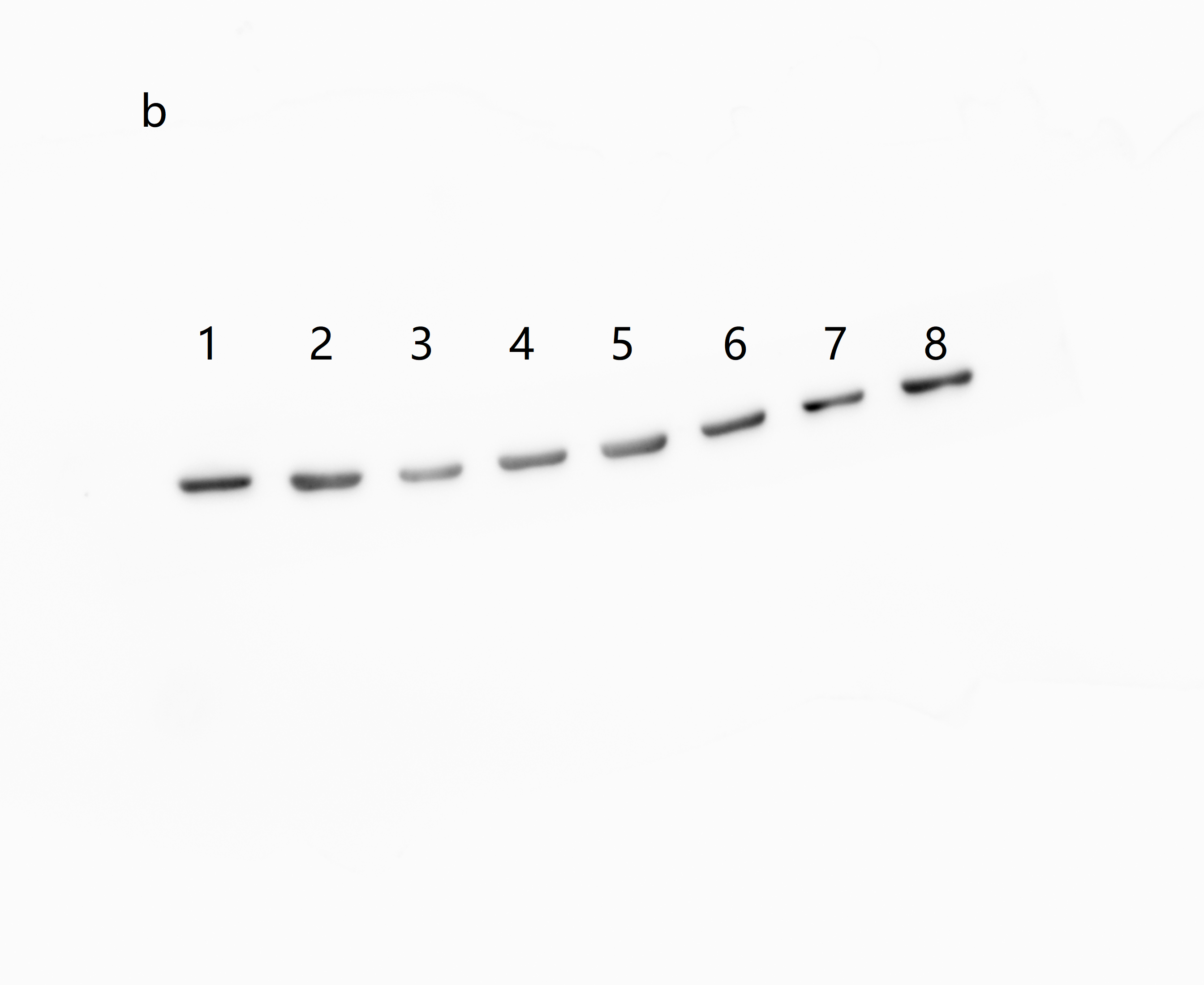


# **Fig 3.** The different compounds, including aspirin (ASA) and atorvastatin (ATR), inhibited NF-κB activation in lung tissue of CVF-induced lung inflammation mice by determining phosphorylated NF-κB p65 protein expression using western blot. Lanes 1-8: control, CVF, PDTC+CVF, ASA+CVF, ATR+CVF, resveratrol+CVF, chlorogenic acid+CVF, icariin+CVF. (**a**) p-p65. (**b**) β-actin. In this manuscript, we only calculated the results on ASA and ATR, and data of other compounds (PDTC, resveratrol, chlorogenic acid, and icariin) were not conducted.

**Fig 4:**


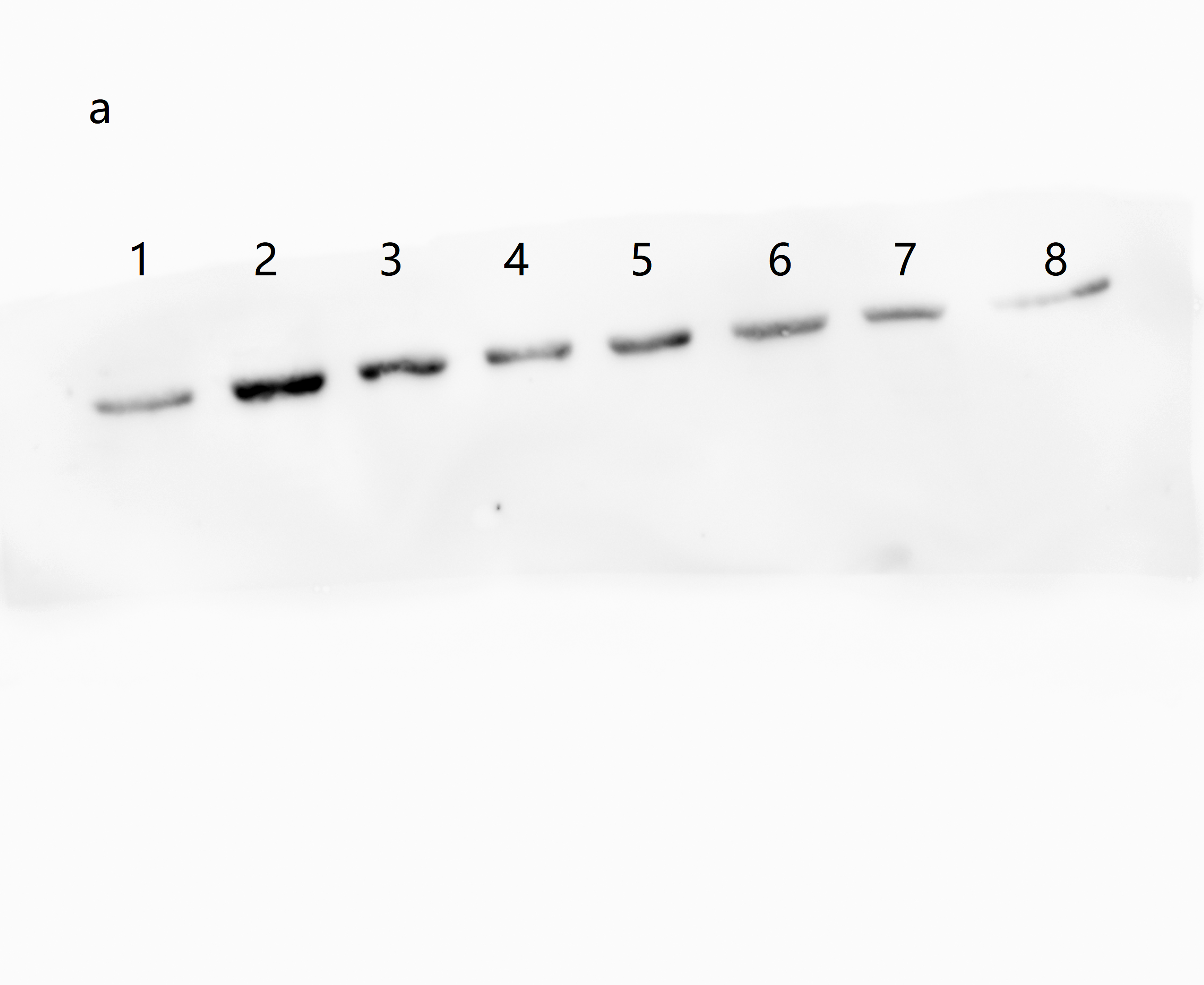


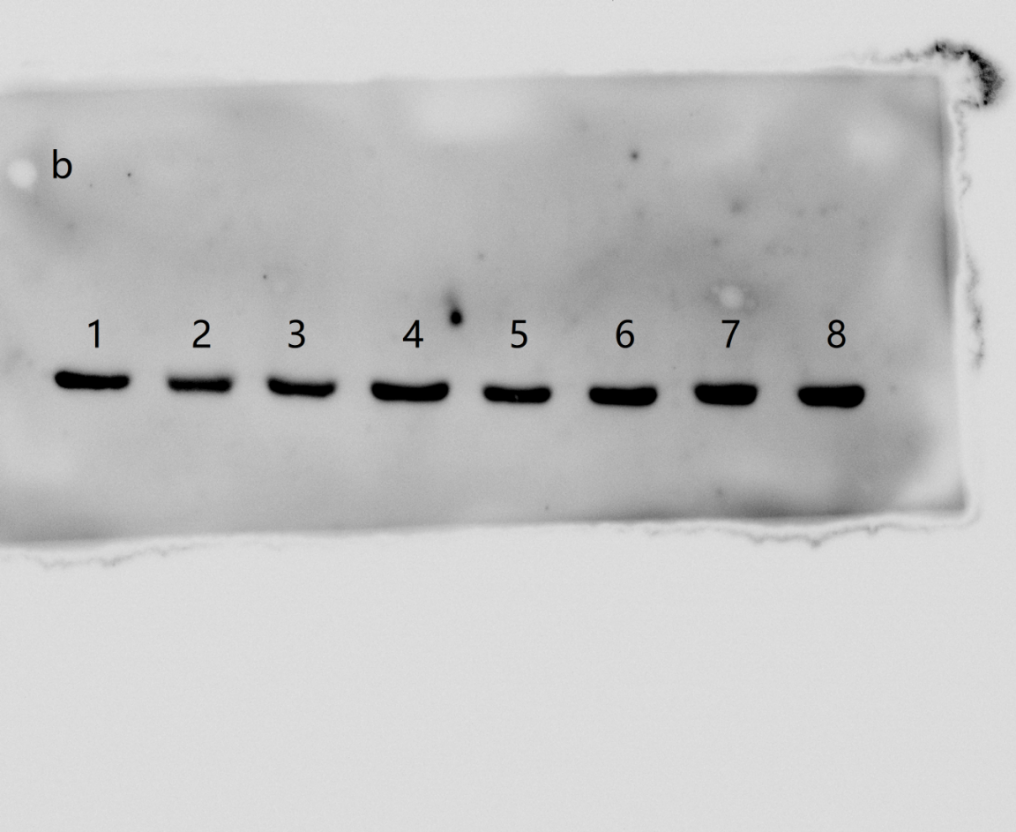


# **Fig 4.** The different compounds, including aspirin (ASA) and atorvastatin (ATR), inhibited NF-κB activation in lung tissue of CVF-induced lung inflammation mice by determining phosphorylated NF-κB p65 protein expression using western blot. Lanes 1-8: control, CVF, PDTC+CVF, ASA+CVF, ATR+CVF, resveratrol+CVF, chlorogenic acid+CVF, icariin+CVF. (**a**) p-p65. (**b**) β-actin. In this manuscript, we only calculated the results on ASA and ATR, and data of other compounds (PDTC, resveratrol, chlorogenic acid, and icariin) were not conducted. The figure 9 as a representative in the manuscript is from the images.

**Fig 5:**


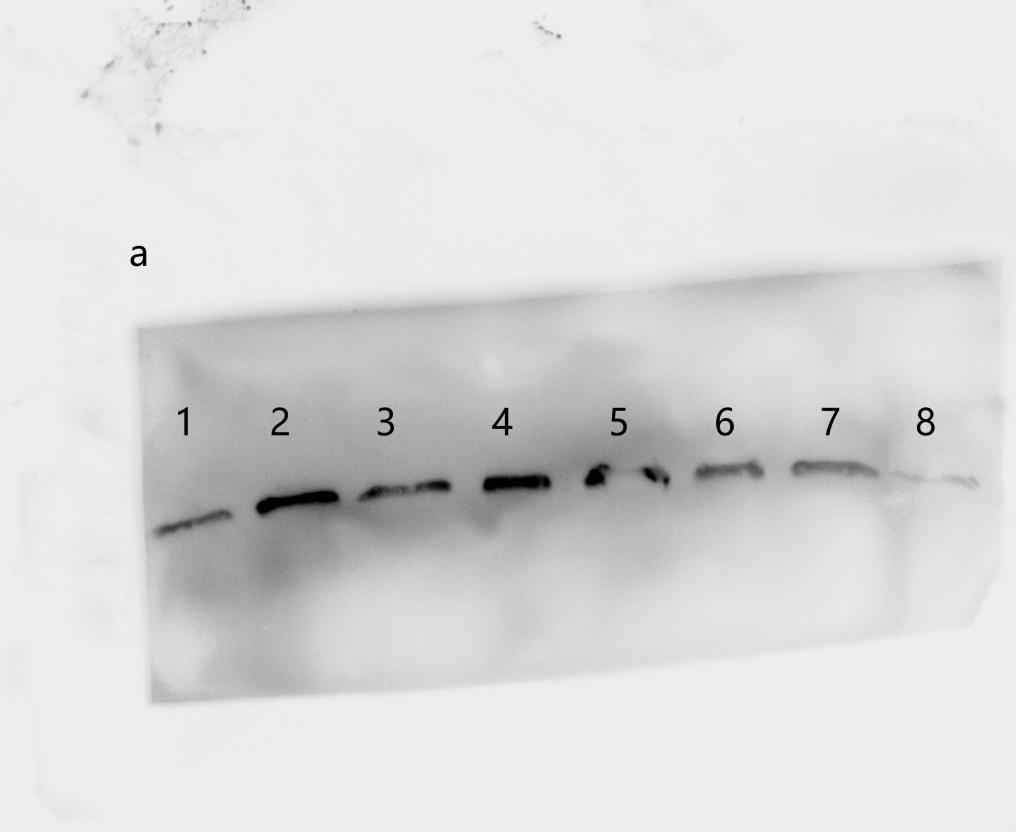


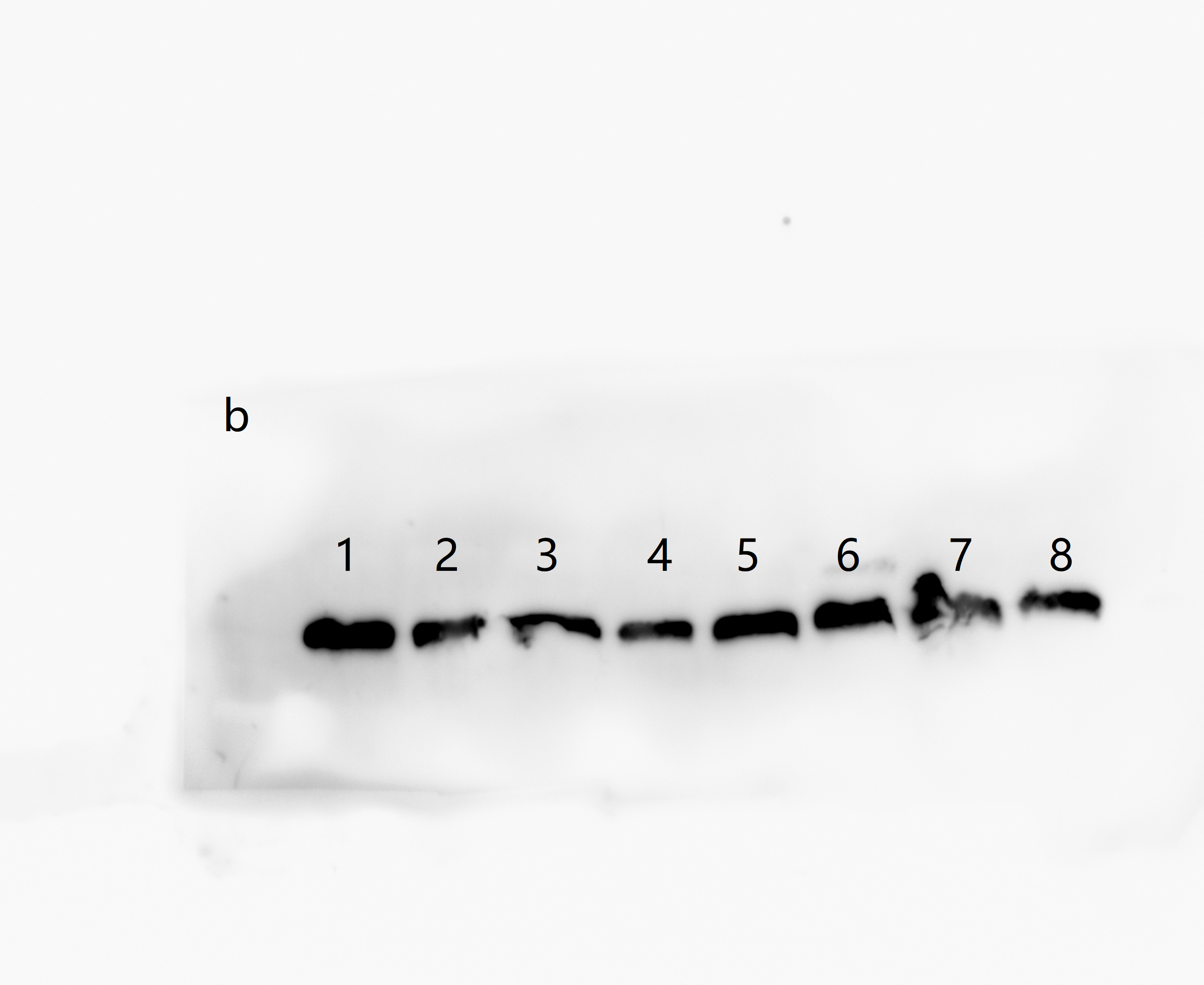


# **Fig 5.** The different compounds, including aspirin (ASA) and atorvastatin (ATR), inhibited NF-κB activation in lung tissue of CVF-induced lung inflammation mice by determining phosphorylated NF-κB p65 protein expression using western blot. Lanes 1-8: control, CVF, PDTC+CVF, ASA+CVF, ATR+CVF, resveratrol+CVF, chlorogenic acid+CVF, icariin+CVF. (**a**) p-p65. (**b**) β-actin. In this manuscript, we only calculated the results on ASA and ATR, and data of other compounds (PDTC, resveratrol, chlorogenic acid, and icariin) were not conducted.

**Fig 6:**


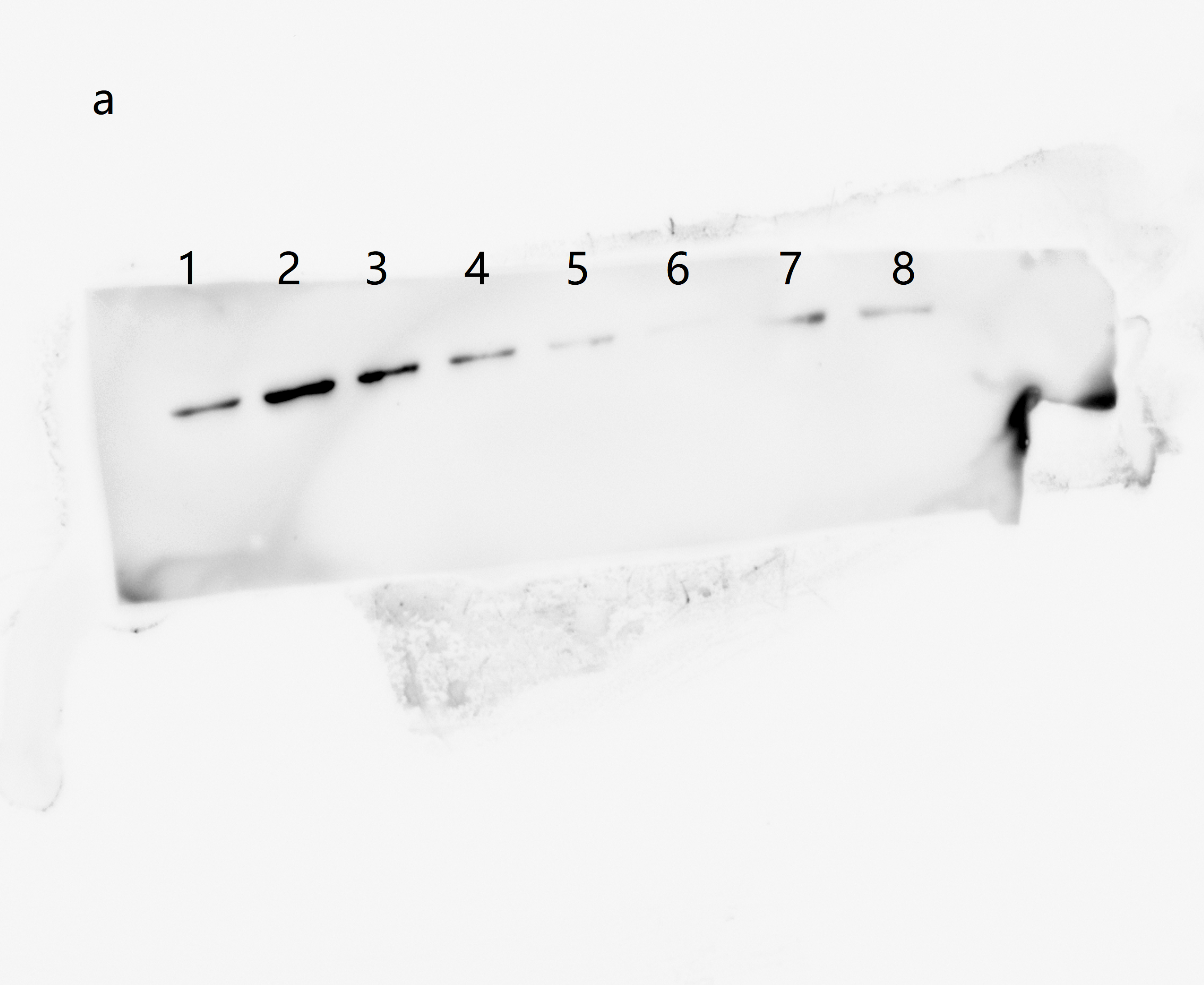

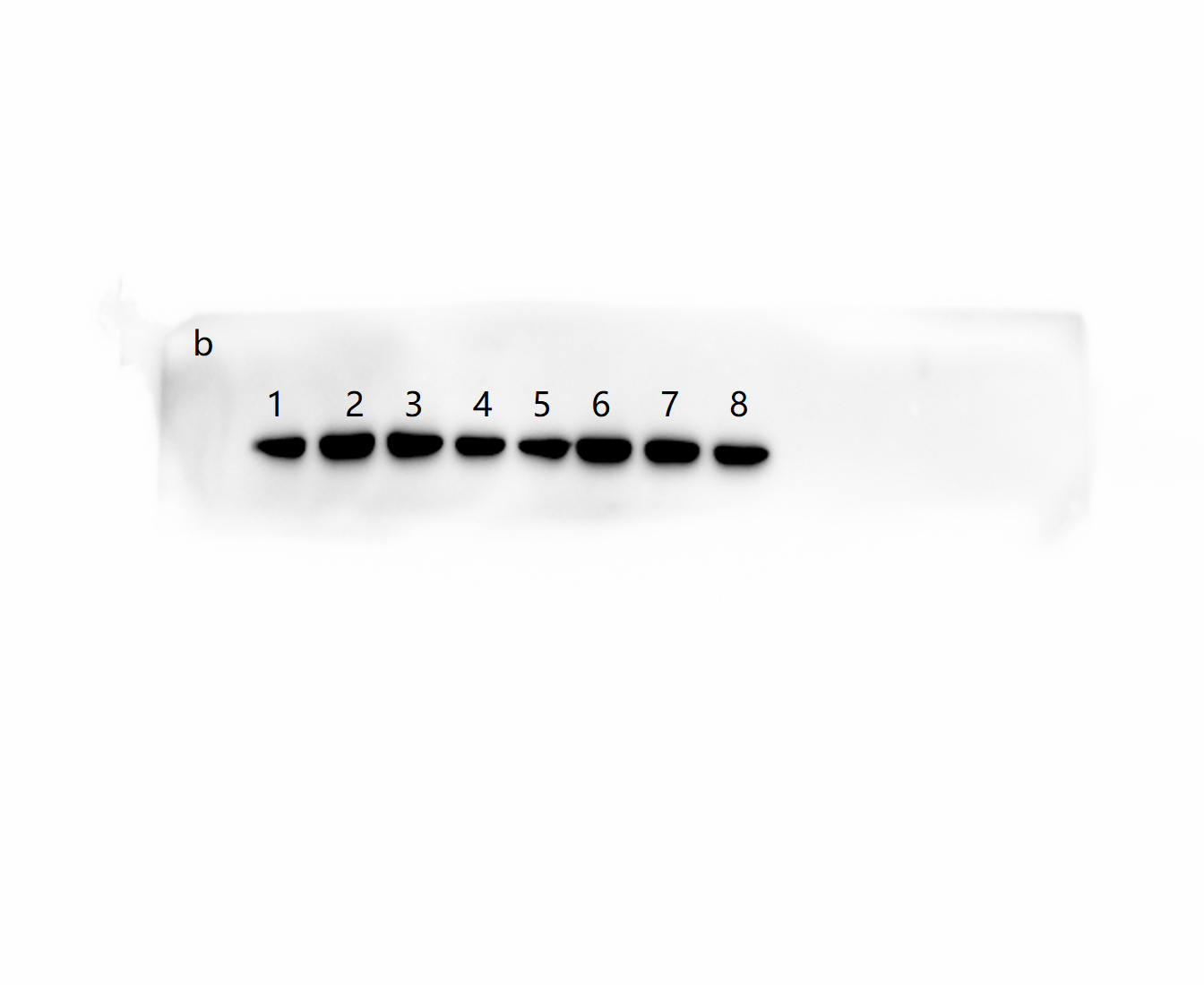


# **Fig 6.** The different compounds, including aspirin (ASA) and atorvastatin (ATR), inhibited NF-κB activation in lung tissue of CVF-induced lung inflammation mice by determining phosphorylated NF-κB p65 protein expression using western blot. Lanes 1-8: control, CVF, PDTC+CVF, ASA+CVF, ATR+CVF, resveratrol+CVF, chlorogenic acid+CVF, icariin+CVF. (**a**) p-p65. (**b**) β-actin. In this manuscript, we only calculated the results on ASA and ATR, and data of other compounds (PDTC, resveratrol, chlorogenic acid, and icariin) were not conducted.

**Fig 7:**


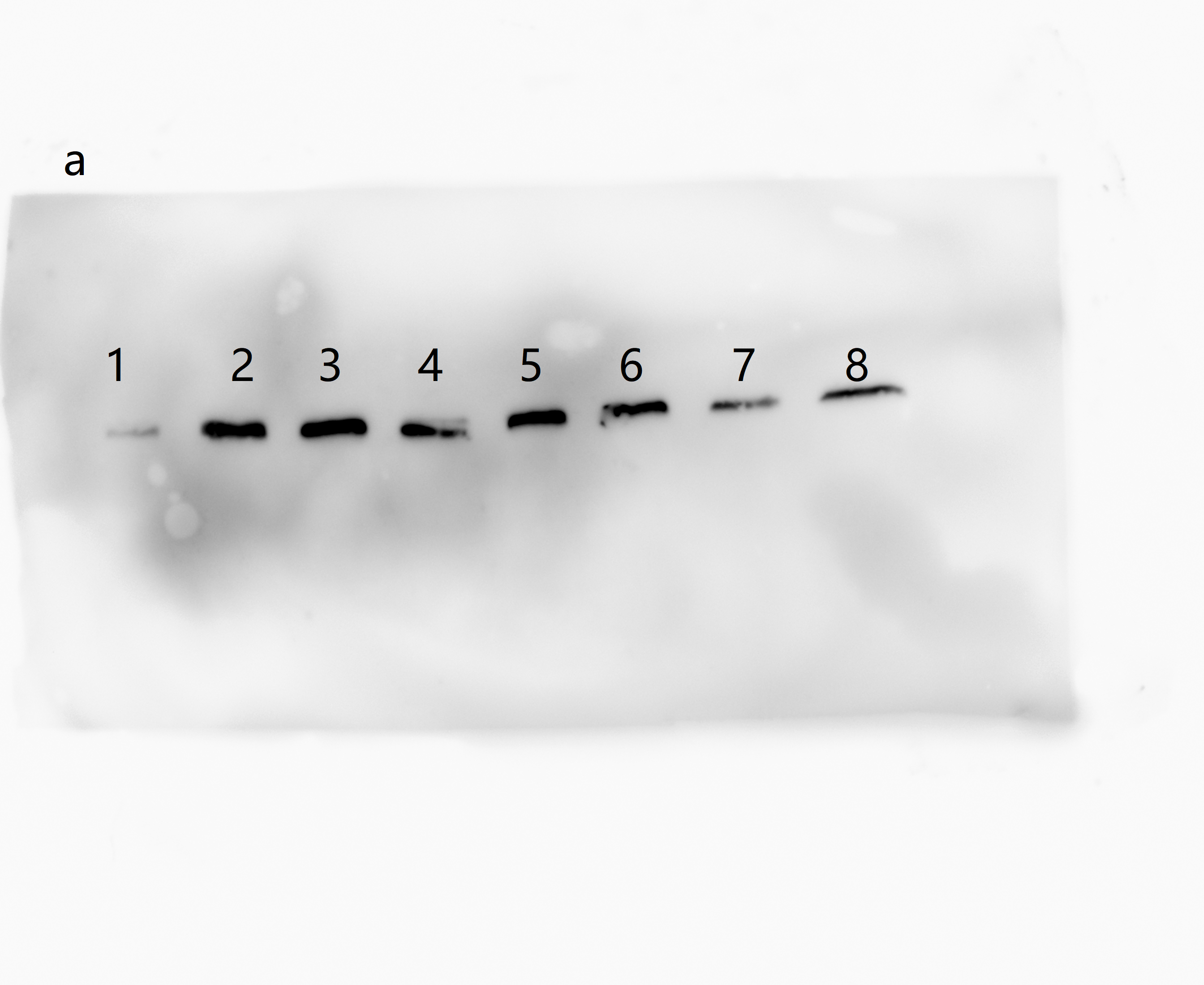


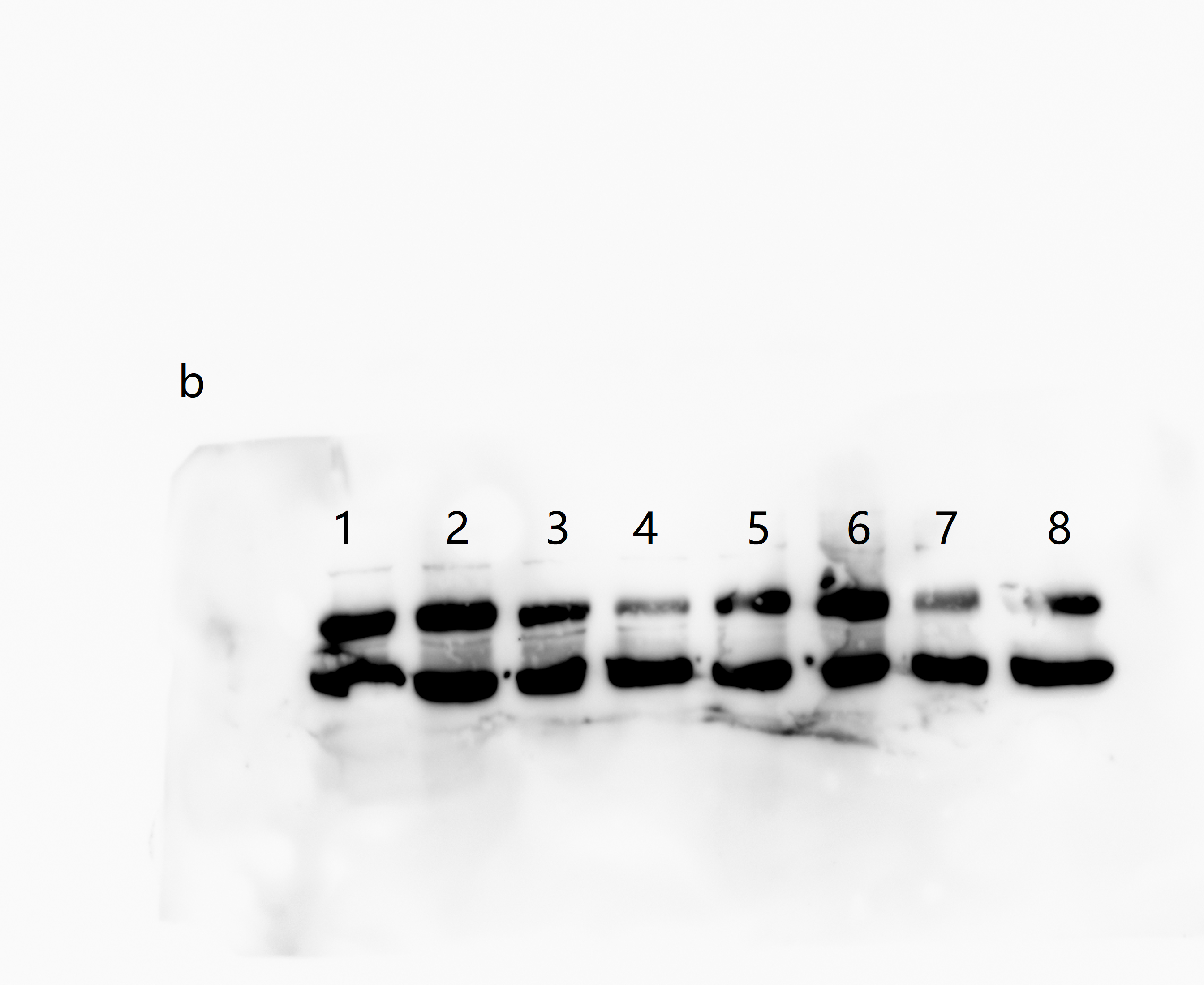


# **Fig 7.** The different compounds, including aspirin (ASA) and atorvastatin (ATR), inhibited NF-κB activation in lung tissue of CVF-induced lung inflammation mice by determining phosphorylated NF-κB p65 protein expression using western blot. Lanes 1-8: control, CVF, PDTC+CVF, ASA+CVF, ATR+CVF, resveratrol+CVF, chlorogenic acid+CVF, icariin+CVF. (**a**) p-p65. (**b**) p65 (upper band) and β-actin (lower band). During the experiment, we first detected the total p65 protein content in the lung tissue, and then detected the β-actin content after washing the blots with TBST. The p65 protein was not completely sealed, so there were two bands in Fig 7b.

**Fig 8:**


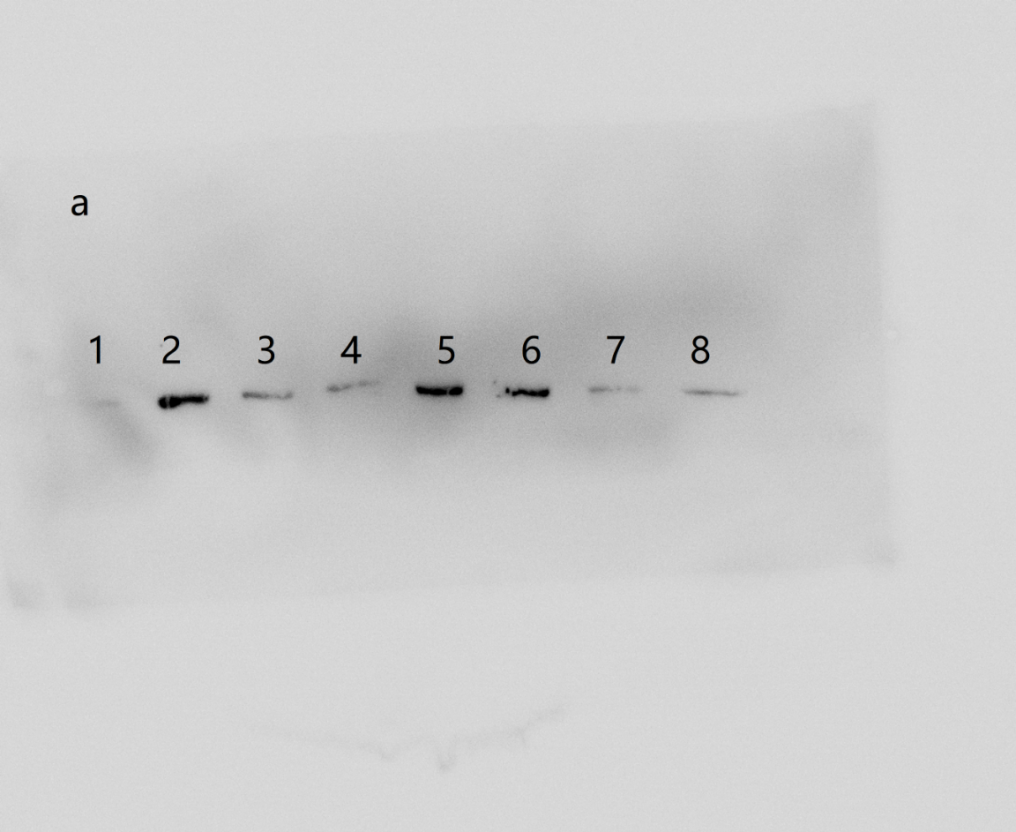


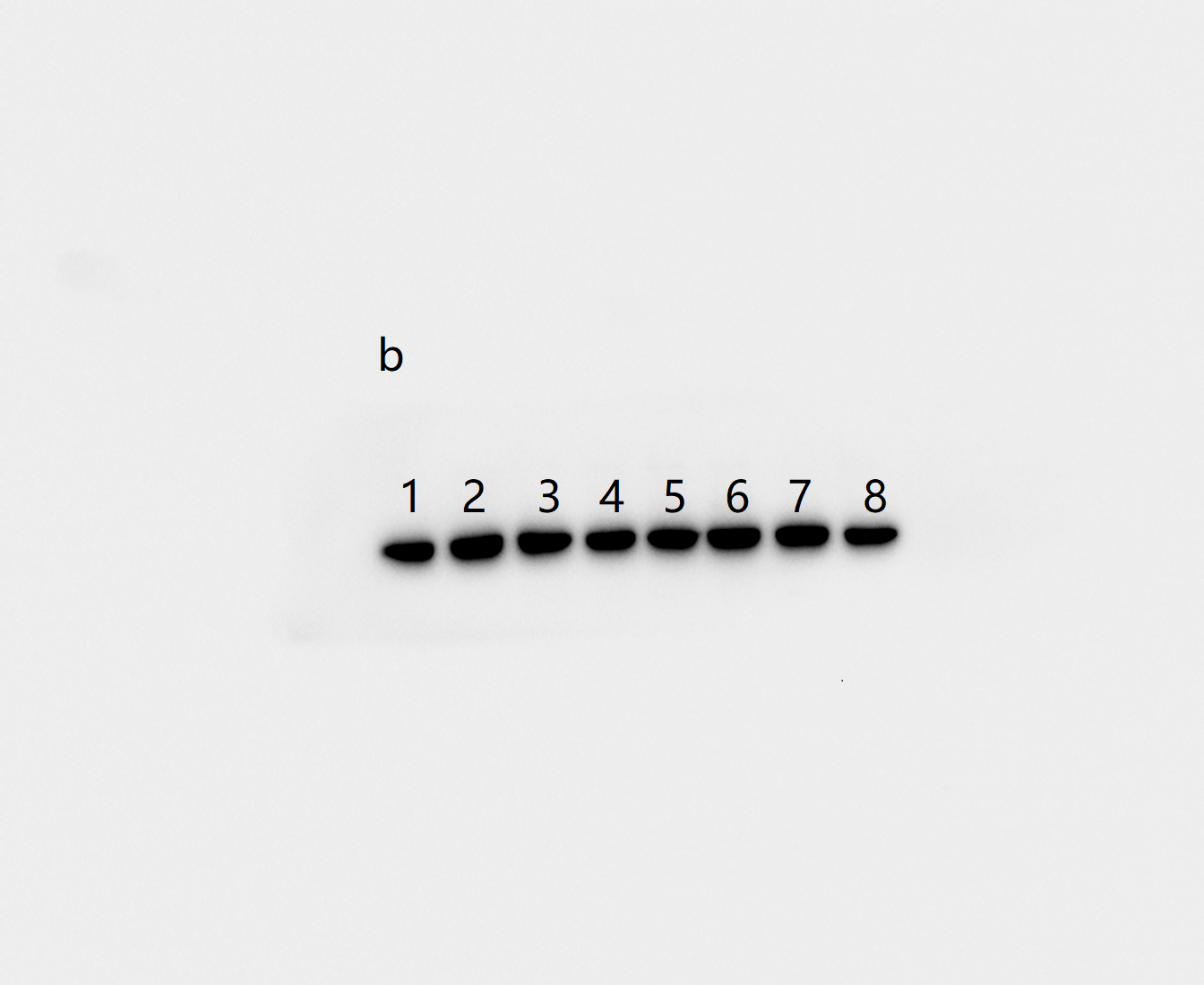


**Fig 8.** The different compounds, including aspirin (ASA) and atorvastatin (ATR), inhibited NF-κB activation in lung tissue of CVF-induced lung inflammation mice by determining phosphorylated NF-κB p65 protein expression using western blot. Lanes 1-8: control, CVF, PDTC+CVF, ASA+CVF, ATR+CVF, resveratrol+CVF, chlorogenic acid+CVF, icariin+CVF. (**a**) p-p65. (**b**) β-actin.
